# Supplementary material for: Role of kif2c, A Gene Related to ALL Relapse, in Embryonic Hematopoiesis in Zebrafish
Source: Int J Mol Sci. 2020 Apr 28;21(9):3127. doi: 10.3390/ijms21093127 (PMC7246619; doi:10.3390/ijms21093127)
Supplement: Supplementary file 1 [file ijms-21-03127-s001.pdf]

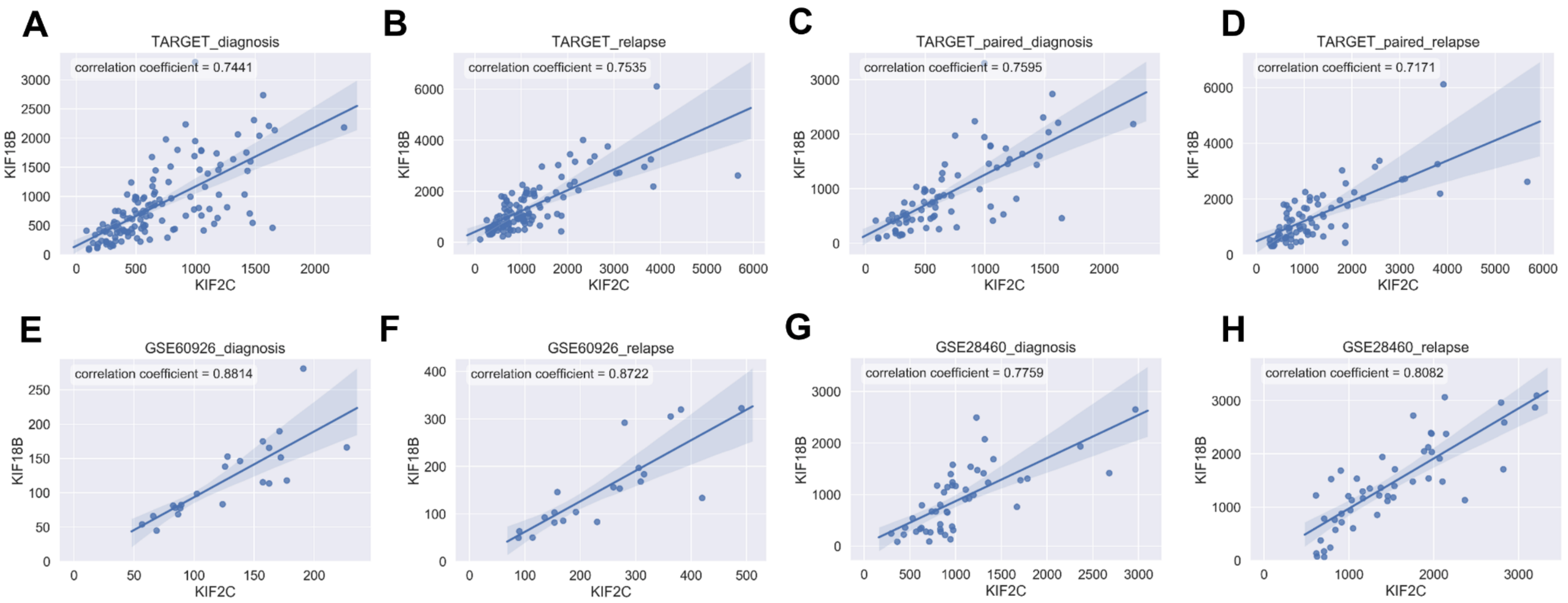

Supplementary Figure 1. Correlation between *KIF2C* and *KIF18B* in (A) the diagnosis sample of the TARGET cohort, (B) the relapse sample of the TARGET cohort, (C) the diagnosis sample of the TARGET_paired sample cohort, the relapse sample of the TARGET_paired sample cohort, (E) the diagnosis sample of the GSE60926 cohort, (F) the relapse sample of the GSE60926 cohort, (G) the diagnosis sample of the GSE28460 cohort, and (H) the relapse sample of the GSE28460 cohort.

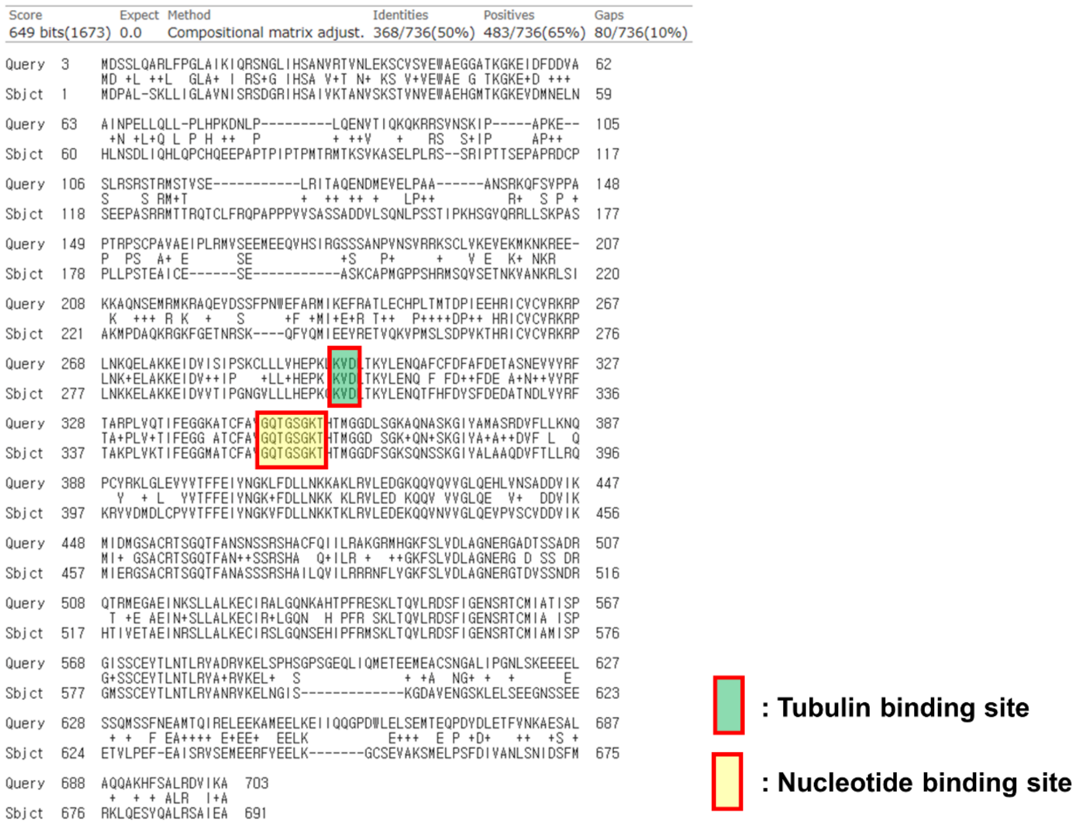

Supplementary Figure 2. KIF2C is evolutionally conserved in vertebrates. Amino-acid sequence alignment of human *KIF2C* (query) and zebrafish *kif2c* (subject) using Pubmed.
